# Infrarenal Abdominal Aortic Cystic Adventitial Disease Detected on Ultrasound and Magnetic Resonance Imaging

**DOI:** 10.3390/diagnostics16040572

**Published:** 2026-02-13

**Authors:** Corrado Tagliati, Alessia Quaranta, Fiammetta Ventura, Fabiola Principi, Enrico Paci

**Affiliations:** 1 AST Ancona, Ospedale di Comunità Maria Montessori di Chiaravalle, Via Fratelli Rosselli 176, 60033 Chiaravalle, Italy; 2 AST Macerata, Cardiologia, Distretto Sanitario di Civitanova Marche, Via Abruzzo, 62012 Civitanova Marche, Italy; alessiaquaranta84@gmail.com; 3 Diagnostic Imaging, Clinical and Interventional Radiology, IRCCS INRCA, 60127 Ancona, Italy; fiam.ventura@gmail.com (F.V.); e.paci@inrca.it (E.P.); 4 AST Ancona, Radiologia, Ospedale Santa Casa di Loreto, Via San Francesco 1, 60025 Loreto, Italy; fabiola.principi@sanita.marche.it

**Keywords:** cystic adventitial disease, aorta, abdominal, asymptomatic, infrarenal, ultrasound, magnetic resonance imaging

## Abstract

Here, we describe a case of an asymptomatic 55-year-old male patient who underwent an abdominal ultrasound examination which showed a para-aortic anechoic lesion without color Doppler signals. A previously performed magnetic resonance imaging examination showed a cystic lesion with features consistent with cystic adventitial disease. To the best of our knowledge, this is the first case of abdominal aortic cystic adventitial disease detected with ultrasound and magnetic resonance images.

**Figure 1:**
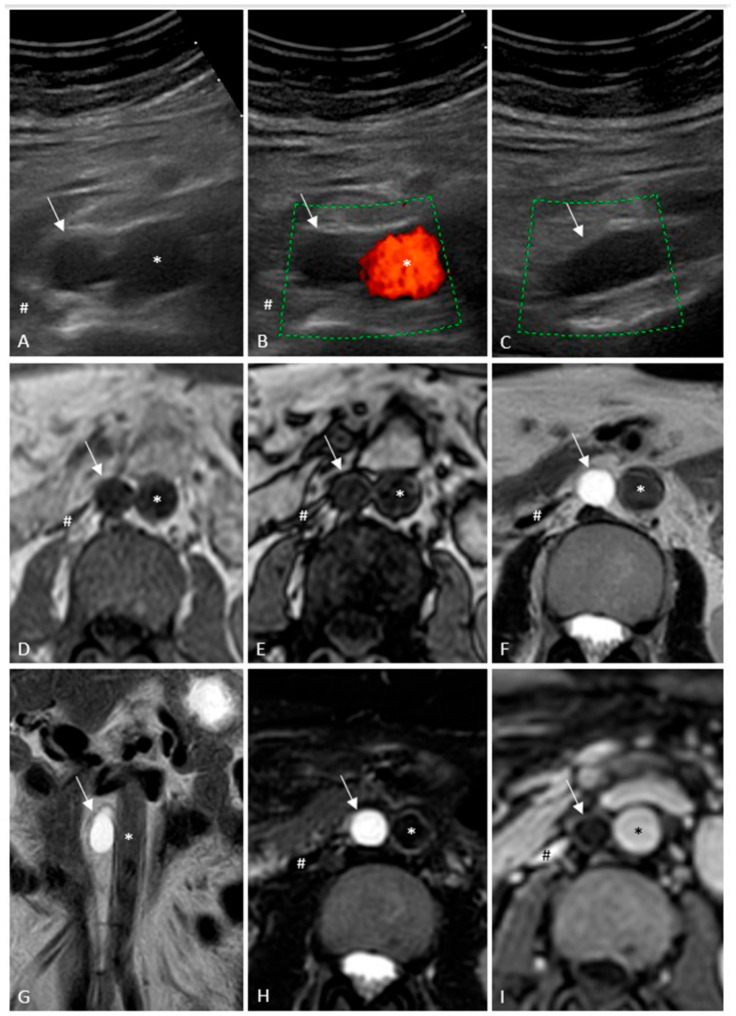
An anechoic lesion of about 1.5 cm in transverse diameter was detected on B-mode abdominal ultrasound (arrow); the lesion seemed to be attached to the right infrarenal abdominal aortic wall, with seamless outer edges (**A**). The abdominal aorta (*) is on the left side of the lesion, and the inferior vena cava (#) is on its right side. No vascularization could be recognized on color Doppler in axial (**B**) and longitudinal (**C**) views; in the latter, the measured cranio-caudal diameter was 2.5 cm. Ultrasound examination was performed to follow-up a hepatic hemangioma, and the aortic finding was incidental. For the same reason, an abdominal magnetic resonance imaging examination performed 2 years before showed a lesion of the same size, hypointense in axial T1 (**D**) and out-of-phase (**E**) sequences, and hyperintense in axial T2 (**F**), coronal T2 (**G**), and axial short tau inversion recovery images (**H**); post-contrast imaging did not show lesion enhancement (**I**). It was not given emphasis on aortic findings at the time of magnetic resonance imaging examination. These features are consistent with cystic adventitial disease, which usually affects the popliteal artery or, more rarely, the femoral and external iliac ones [1,2,3,4,5,6,7,8]. A few cases of radial artery involvement were reported, and only one previously published material was about the abdominal aorta [9,10,11,12]. The latter reported an approximately 54-year-old female patient who was treated surgically for a suspected aneurysm of the abdominal aorta, but upon surgical exploration, a cystic lesion was detected and resected [12]. In order to exclude concomitant leg disease, our patient underwent a lower-extremity arterial Doppler, which was negative. Cystic adventitial disease is a possible cause of claudication, as it can reduce the adjacent arterial lumen; however, in our case, no evident luminal aortic diameter reduction was detectable; in fact, our patient was completely asymptomatic and he did not have vascular risk factors or previous trauma. Four theories about cystic adventitial disease development are reported in the literature, in particular: de novo mucinous degeneration theory; trauma or repetitive vessel stretching and distortion theory; articular or synovial theory due to repetitive microtrauma to the adjacent joints, which results in tracking of the synovial fluid to the adjacent arterial vasculature; developmental theory related to mucin-secreting mesenchymal cells placed in the adventitia of the vessels during embryogenesis. The last two theories enjoy the most support [1,2,5,13]. However, in our case, causal interpretation is not possible. The first reported case of cystic adventitial disease involved an external iliac artery and was described by Atkins and Key in 1947, and, as written before, only one case pertaining to the abdominal aorta was reported, by Kitzis et al. in 1983 [12,14]. Therefore, to the best of our knowledge, this is the first case of an abdominal aortic cystic adventitial disease detected with ultrasound and magnetic resonance images. However, its value is purely descriptive and does not allow for generalizable conclusions regarding the prevalence, incidence, or predictive value of imaging. We think that it is important to know the imaging features of this disease in order to avoid misinterpretations during abdominal ultrasound or magnetic resonance imaging examinations. Previously published articles described the value of magnetic resonance imaging, which is considered the noninvasive modality of choice in this disease [15,16]. Moreover, in our case, diagnosis remains presumptive as surgical excision was not performed and, presumably, it will not be performed if the patient will remain asymptomatic with a stable lesion in the next few years. However, a vascular surgeon visit is scheduled in four months to decide the proper management.

## Data Availability

The original contributions presented in this study are included in the article material. Further inquiries can be directed to the corresponding authors.
